# Effect of Hydrophobic Interactions on Lower Critical Solution Temperature for Poly(*N*-isopropylacrylamide-co-dopamine Methacrylamide) Copolymers

**DOI:** 10.3390/polym11060991

**Published:** 2019-06-04

**Authors:** Alberto García-Peñas, Chandra Sekhar Biswas, Weijun Liang, Yu Wang, Pianpian Yang, Florian J. Stadler

**Affiliations:** 1 College of Materials Science and Engineering, Shenzhen Key Laboratory of Polymer Science and Technology, Guangdong Research Center for Interfacial Engineering of Functional Materials, Nanshan District Key Laboratory for Biopolymers and Safety Evaluation, Shenzhen University, Shenzhen 518055, China; alberto@szu.edu.cn (A.G.-P.); chandra123@szu.edu.cn (C.S.B.); weijunliang1116@hotmail.com (W.L.); one.wangyucoak@outlook.com (Y.W.); 2 Key Laboratory of Optoelectronic Devices and Systems of Ministry of Education and Guangdong Province, College of Optoelectronic Engineering, Shenzhen University, Shenzhen 518060, China; 3 Department of Management, Shenzhen University, Shenzhen 518060, China

**Keywords:** *N*-isopropylacrylamide, lower critical solution temperature, thermoresponsive polymers, hydrophobic interactions, statistical modeling

## Abstract

For the preparation of thermoresponsive copolymers, for e.g., tissue engineering scaffolds or drug carriers, a precise control of the synthesis parameters to set the lower critical solution temperature (LCST) is required. However, the correlations between molecular parameters and LCST are partially unknown and, furthermore, LCST is defined as an exact temperature, which oversimplifies the real situation. Here, random N-isopropylacrylamide (NIPAM)/dopamine methacrylamide (DMA) copolymers were prepared under a systematical variation of molecular weight and comonomer amount and their LCST in water studied by calorimetry, turbidimetry, and rheology. Structural information was deduced from observed transitions clarifying the contributions of molecular weight, comonomer content, end-group effect or polymerization degree on LCST, which were then statistically modeled. This proved that the LCST can be predicted through molecular structure and conditions of the solutions. While the hydrophobic DMA lowers the LCST especially the onset, polymerization degree has an important but smaller influence over all the whole LCST range.

## 1. Introduction

The research on smart polymers is growing, owing to new advances of the scientific community as well as current and future applications. Some thermoresponsive polymers present an extraordinary sensitivity to external stimuli related to temperature, pH, light, or solvents, among others. These properties opened new interesting applications regarding to scaffolds or drug carriers [1,2,3,4].

The use of poly(*N*-isopropylacrylamide) (PNIPAM) as a water-soluble polymer is extended along numerous publications because its lower critical solution temperature (LCST) is near human physiological temperature (around 33 °C) [5,6]. Generally, a homogenous and transparent solution is observed below LCST, but higher temperatures lead to insolubility of the polymer in water. This transition can be explained based on hydrogen bonding and hydrophobic interactions between polymer and water, whose balance is temperature dependent [7,8,9].

Various ways for varying LCST behavior are used in practice—such as adding comonomer, modification of the end group or introduction of additives [10]. Block copolymers (vs. random copolymers) can provide a second phase transition promoting new features to the final material and according to the monomers involved, but synthesis can require more steps than making other structures.

Generally, the preparation of random copolymers is used as a valuable method for adding new functionalities or increasing the range of properties. In this way, bioinspired materials grow a great interest owing to simulate the living tissues very useful for developing specific properties and getting non-invasive therapies. For instance, the use of catechol groups was inspired by marine mussels, which produce proteinaceous adhesive materials for attaching themselves to rocks in the intertidal zone [11]. The catechol groups show a great versatility in terms of applications such as biosensors or biomedicine [12,13,14,15,16,17]. Moreover, the use of dopamine methacrylamide (DMA) as a comonomer is suitable due to its main chain structure being similar to *N-*isopropylacrylamide (NIPAM) [18].

The introduction of reversible addition-fragmentation chain transfer (RAFT) polymerization allowed getting custom polymers due to a higher control on the final molecular features, namely polydispersity and molecular weight [19,20]. Additionally, this potential method is convenient for preparing copolymers, whose properties, such as the LCST, can be tailored to the specific necessities. At present, the influence of some parameters on LCST is clear but there are still many inconsistences related to the influence of molecular weights and end-group effects, among others. Important information associated with LCST transitions is missed, which could partially explain those influences. In general, all research works are focused on a single temperature value for defining the LCST, but information about the range of temperature or time of this process is obviated. Thus, a complete evaluation of LCST transition could be very useful for this purpose.

There are different procedures for detecting LCST as turbidimetry, calorimetry, proton nuclear magnetic resonance, rheology, or dynamic light scattering, which provide rich information. Specifically, calorimetric analysis can provide an estimate of the number of hydrogen bonds involved on this process [21]. In terms of applications, slow LCST transitions could be desired for a specific slow drug release, while fast LCST transitions could be useful for biosensors.

This work is focused on the preparation of several random copolymers, where molecular weight and comonomer composition were accurately varied in line to investigate the influence of these parameters on LCST transition. The use of different characterization methods (calorimetry analysis, UV-visible spectroscopy, and rheology) allowed for detecting the sudden change from hydrophilic to hydrophobic behavior, i.e., the LCST. Those changes will be modeled with a statistical regression analysis to check for the diverse contributions of parameters involved in LCST.

## 2. Experimental

### 2.1. Materials

Sodium borate (99%, Macklin Reagent Company, Shanghai, China, sodium bicarbonate (99.8%, Macklin Reagent Company, Shanghai, China), 3,4-dihydroxyphenethylamine hydrochloride (98%, Sigma-Aldrich, Hamburg, Germany), azobisisobutyronitrile (99%, Aladdin, Shanghai, China) were used without pretreatment. Diverse solvents, as tetrahydrofuran (99.9%, Aladdin, Shanghai, China) and *N*,*N*-dimethylformamide (99%, Aladdin, Shanghai, China), were distilled under sodium and calcium chloride with nitrogen bubbling. *N*-Isopropylacrylamide (98%, Aladdin, Shanghai, China) was recrystallized from a mixture of hexane and benzene (65:35). The RAFT-agent I-phenylethyl phenyldithioacetate was prepared according to literature [22].

### 2.2. Synthesis of Dopamine Methacrylamide (DMA)

The preparation of dopamine methacrylamide (DMA) was carried out according to the procedure of Glass et al. [23]. The resulting powder was purified under a solution of methyl acetate (40 mL), and subsequently, the obtained monomer was precipitated in 600 mL hexane.

### 2.3. Synthesis of Random Poly(NIPAM-co-DMA) Copolymers

The different RAFT copolymerizations were carried out under inert conditions in dry Schlenk tubes where *N-*isopropylacrylamide (NIPAM) and DMA were placed. Different amounts of azobisisobutyronitrile (AIBN) and I-phenylethyl phenyldithioacetate (PEPD) were used as initiator and RAFT agent, respectively. Then, *N*,*N*-dimethylformamide was added as a solvent and the mixture was kept in a nitrogen environment using a Schlenk system. Subsequently, Schlenk tubes were placed in a thermostatted bath at 70 °C for 48 h, and reactions were stopped by freezing in liquid nitrogen.

The random copolymers (SIScheme 1) were purified three times by precipitation in diethyl ether, and finally, samples were dried under vacuum for 48 h. The samples were stored at room temperature.

Samples were denominated according to the DMA content and the molecular weight. For example, C5M4000 is associated with a DMA content of 5.5 mol % and a molecular weight of 4300 g/mol. The NIPAM homopolymers were prepared under similar conditions and used as reference. These were exclusively denominated according to the molecular weight.

Aqueous polymer solutions with 4, 8, 10, and 15 wt % concentrations were prepared and stored in a refrigerator for 12 h to ensure the complete dissolution before LCST analysis.

### 2.4. Analytical Methods

#### 2.4.1. Nuclear Magnetic Resonance

The DMA content was estimated by proton nuclear magnetic resonance for the diverse copolymers (Table 1). Proton nuclear magnetic resonance spectra were recorded with an AVANCE III 600 MHz spectrometer (Bruker, Switzerland) at 25 °C using deuterated DMSO as a solvent [24].

#### 2.4.2. Gel Permeation Chromatography (GPC)

The molecular weights and polydispersities (PDI) were estimated by size exclusion chromatography in a Beijing Wenfen LC98IIRI (Beijing, China; Table 1). Two polystyrene gel columns (Shodex, KD-803 and KD-806, Detector: RI-201H) were used, and tetrahydrofuran was selected as a solvent. The measurements were carried out at 40 °C and at a flow rate of 1 mL/min. Narrow molecular mass distribution polystyrene standards were used to calibrate the experiments. In our previous paper [11], we found that a polystyrene-calibrated GPC produces ca. 35% lower values for copolymers with 2.5 mol% and 5 mol% DMA in comparison to vapor pressure osmometry—an absolute method for the determination of molar mass M_n_. Hence, while the PS-calibrated values are not absolute, they can be considered to be good approximations of the absolute values.

#### 2.4.3. Thermogravimetric Analysis

Thermo-gravimetric (TGA—TA Instruments Q50, New Castle, PA, USA.) analysis was used for the confirmation of the real sample concentration in water. The polymer content was evaluated in a broad range of temperature defined between 25 and 250 °C at a heating rate of 20 °C/min in nitrogen atmosphere.

#### 2.4.4. Differential Scanning Calorimetry (DSC)

The calorimetric analysis was performed in a Q200 Differential Scanning Calorimeter (TA Instruments, New Castle, PA, USA) with a cooling system. The sample weight was around 5 mg and the machine was calibrated with different standards.

The temperature of the phase transition was analyzed at a heating rate of 5 °C/min in the range of 0–60 °C for the diverse polymer solutions in water in sealed crucibles to avoid evaporations. Three scans were performed for each sample to evaluate the reproducibility of results. The resulting data were normalized, and the baseline was corrected with another experiment performed in the same conditions with the same amount of pure water.

Moreover, the glass transition temperature was estimated by calorimetric analysis. The experiments were carried out at 20 °C/min with a flow of 40 mL/min nitrogen from 20 to 250 °C.

#### 2.4.5. UV-Visible Spectroscopy

The thermal transitions were studied using a UV-vis spectrophotometer, PerkinElmer UV/VIS Lambda 365 (Seoul, Korea), with temperature control. The transmittance of the diverse polymer solutions in water was tested in a wide range of temperatures from 15 to 40 °C with a heating rate of 1 °C/min at 500 nm. Finally, the cloud points were calculated at 50% transmittance for each polymer solution.

#### 2.4.6. Rheological Measurements

LCSTs were also evaluated by rheological measurements with a cone plate geometry (15 mm/2°) where temperature dependence was evaluated with *q* = 1 K min^−1^, ω = 0.16 s^−1^, and γ_0_ = 2–5% under linear conditions in an Anton Paar MCR 302 rheometer (Graz, Austria) in a humidity saturated atmosphere.

### 2.5. Statistical Modeling

To analyze the relationship between diverse values associated with the LCST (onset temperatures T_onset_, peak temperatures T_peak_, cloud temperatures T_cloud_, offset temperatures T_onset_, and the LCST ranges LSCT_range_), obtained from calorimetry and turbidimetry, and the characteristics of the polymeric solutions (comonomer content, polymerization degree, and the polymer concentration in water) a regression analysis was done by the SPSS software.

## 3. Results

### 3.1. Synthesis and Molecular Characterization

The DMA content was estimated by ^1^H-NMR for the copolymers in d_6_-DMSO. The proton signals were elucidated in relation to the data reported in the literature where a good equivalence was observed (Figure S1) [11]. The integration of NH-CH-(CH_3_)_2_ (δ = 3.85 ppm) and benzene signals (δ = 6.52–6.70 ppm) allowed to estimate the content of DMA (Figure S2). The DMA contents are displayed in Table 1 where C5M4000, C5M18000, and C5M24000 samples show similar values.

**Figure 1:**
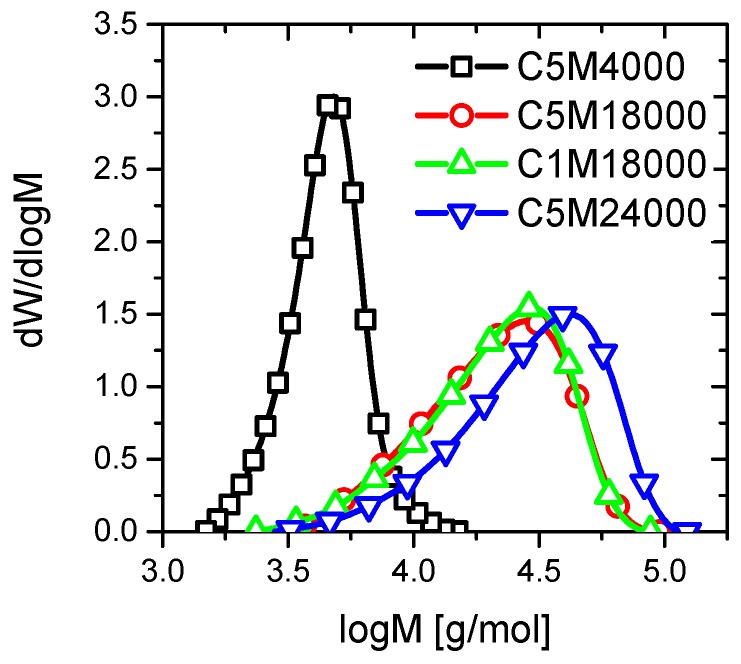
Molecular weight distribution profiles obtained from GPC curves for the copolymers with different comonomer content and molecular weights.

The GPC results show a narrow molar mass distribution (PDI) for all materials as can be deduced from PDI values in Table 1, together the different molecular weights (M_n_). The polydispersity values can be explained through the benefits of RAFT polymerization. Figure 1 shows unimodal curves of the molecular weight distribution profiles for the copolymers of this research work. Similarities between C5M18000 and C1M18000 curves allow for getting a great uniformity between size and molecular weight of polymeric chains. Therefore, these features can be essential for excluding the influence of polydispersity on the LCST behavior of C5M18000 and C1M18000 samples (Figure 1).

A low amount of RAFT agent during polymerization could justify a slight increment into the polydispersity of C5M24000, C1M18000, and C5M18000 samples (Table 1) because control agents provide narrow polydispersities [25]. Nevertheless, the polydispersities are relatively narrow for all materials owing to RAFT polymerization vs. traditional free radical polymerization.

### 3.2. LCST Behavior

The preparation of diverse aqueous solutions (4, 8, 10, and 15 wt %) was carried out in relation to studying the LCST behavior for the copolymers by conventional calorimetry, turbidimetry (UV-visible spectroscopy), and rheology. The real polymer concentration in water was confirmed by TGA analysis, where as expected a clear equivalence was observed between theoretical concentrations of solutions and the data estimated from TGA-curves (Figure S2).

**Figure 2:**
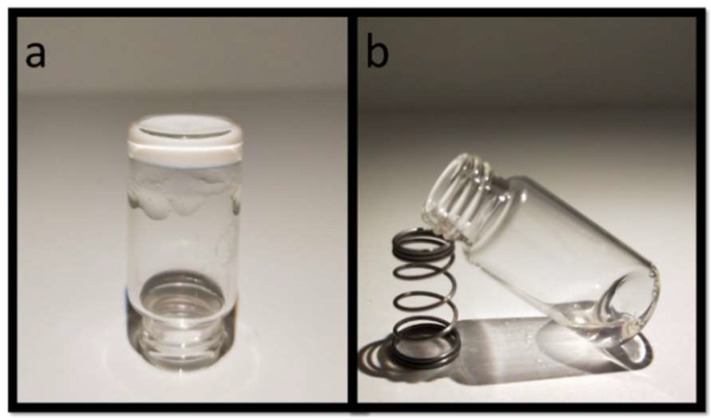
Polymer solution in water (C5M24000, c = 10 wt %) above LCST (**a**) and below LCST (**b**).

The coil to globule transition can be easily observed visually (Figure 2), where a sample below LCST is a transparent aqueous solution due to hydrogen bonds between polymer–water (Figure 2b), whereas the polymer–polymer interactions increase over LCST and the hydrogen bonds are broken, consequently polymer globules are formed and the sample becomes solid and opaque (Figure 2a) [26]. Moreover, the polymers are shown to have statistical DMA distributions—as expected—because block copolymers tend to form hairy micellar solutions in selective solvents, which likely are often turbid (Figure 2a) [27,28,29,30,31]. Furthermore, it is clear from Figure 2 that the DMA is not oxidized, as this would lead to lead to catechol tanning, which turns the sample dark red after an initial pink hue at very low oxidation levels.

### 3.3. Influence of DMA on LCST

**Figure 3:**
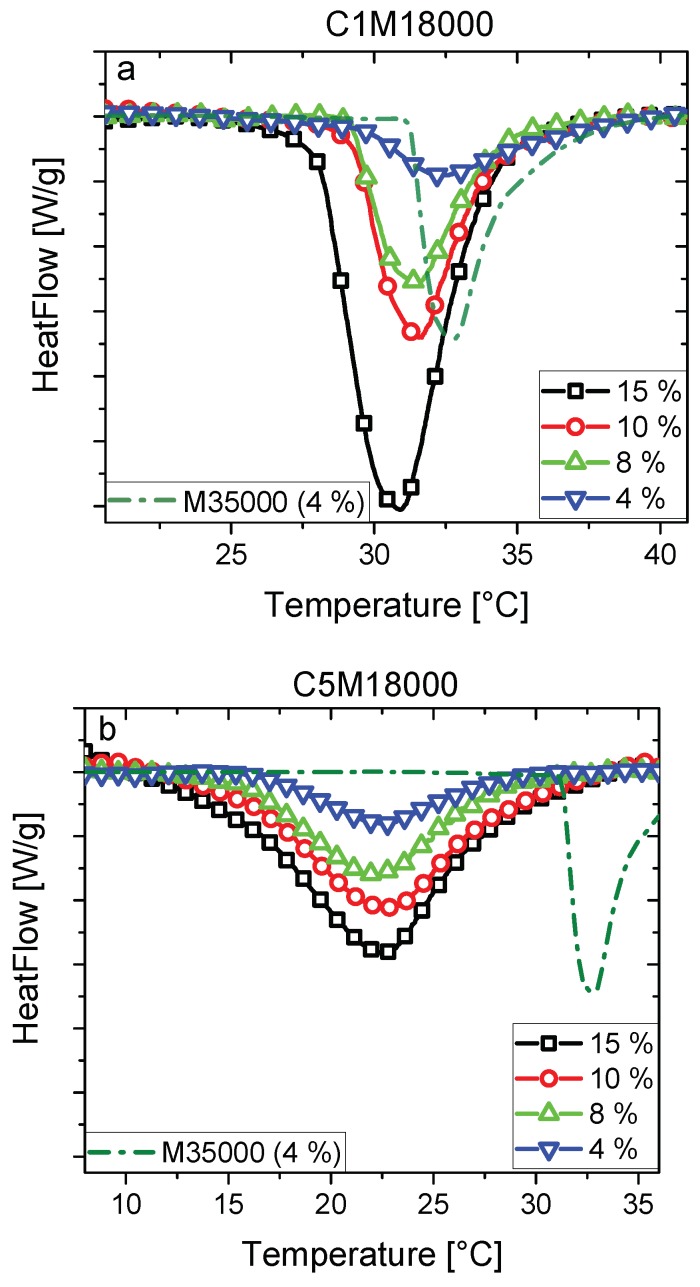
DSC endotherms of aqueous solutions (4, 8, 10, and 15 wt %) for C1M18000 (**a**) and C5M18000 (**b**) samples. The results were compared with M35000 homopolymer (c = 4 wt %, dash-dot green line).

The different endothermic processes of the diverse polymer solutions (4, 8, 10, and 15 wt %) were carried out by calorimetric analysis for C1M18000 and C518000 (Figure 3). The differences between both polymers, whose comonomer amount was varied, are evident from the thermograms.

Firstly, the peak temperatures of C1M18000 are over 30 °C, whilst LCSTs are found below 25 °C for C5M18000. A higher content of DMA in C5M18000 could explain the decrease of LCST regarding to C1M18000. Other influences such as polydispersity or end-group effect were dismissed according to the GPC results. In general, the incorporation of the hydrophobic comonomers reduces the LCST whereas hydrophilic comonomers increase the LCST values [32,33,34,35,36]. As the hydrophobicity of DMA is higher than NIPAM, the LCST tends to decrease when comonomer content increases [11]. The homopolymer showed a slightly higher LCST than C1M18000 due to low DMA content and high polymerization degree. A possible end-group effect caused by the differences in molecular weight between C1M18000 and M35000 was rather small, as will be discussed later. C5M18000 exhibited lower LCST due to the higher comonomer amount, increasing its hydrophobicity.

Secondly, C1M18000 has sharp and narrow transitions for all solutions with regard to the endotherms associated with C5M18000. Furthermore, C1M18000 seems to show a better thermal sensitivity [10] than C5M18000 as endotherms in Figure 3 show. Higher hydrophobic interactions could promote longer transitions when the DMA content is increased, i.e., lower thermal sensitivity such as C5M18000 endotherms displayed (Figure 3b). Generally, the LCST transition defines the temperature where the hydrogen bonds are breaking [37] as C1M18000 endotherms clearly show (Figure 3a). Nevertheless, C5M18000 displays another effect during the LCST transition, i.e., hydrophobic DMA could partially slow down the LCST leading to wider transitions where LCST occurs.

**Figure 4:**
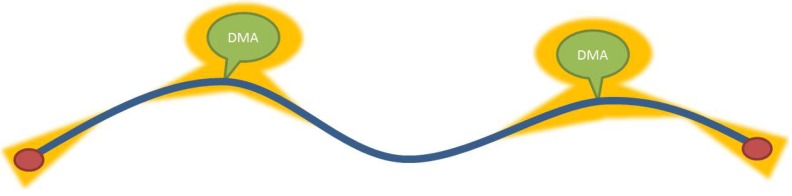
Scheme of the hydrophobicity fluctuations along the chain.

Thirdly, DSC shows clearly transitions for C1M18000 in comparison to C5M18000, but less clear than for M35000. Generally, the calorimetric analysis provides information about the LCST, i.e., these endotherms allow for determining the number of hydrogen bonds broken during the LCST transition [21]. The presence of hydrophobic DMA interferes with the LCST transition, and consequently, the transition width increases (Figure 3b). This can be understood through the fact that the DMA monomer itself in the chain is hydrophobic enough that it would render the DMA homopolymer water-insoluble. Thus, in the vicinity of the DMA monomer, the hydrophobicity was higher ((Figure 4, green circles), the blue line stands for NIPAM monomers, red circles are the RAFT-end groups and the green circles are the hydrophobic dopamine rings of DMA. The orange shading stands for the local hydrophobicity). Obviously, this effect becomes more pronounced, when 2 DMA monomers are adjacent to each other, at 1–6 mol % DMA content is rather unlikely. As a consequence, the chain has to be considered to have hydrophobicity variation along the chain, depending on the local comonomer distribution. Furthermore, also the end groups play a role, as their hydrophobicity is even greater than DMA (Figure 4, red circles), which leads to the same hydrophobicity fluctuations.

On the other hand, C1M18000 endotherms show a clear LCST dependence with polymer concentration in water, i.e., decreasing peak temperatures as polymer concentration increases probably because a dilute medium reduces the polymer–polymer interactions, and thus hydrogen bonds could be stronger.

**Figure 5:**
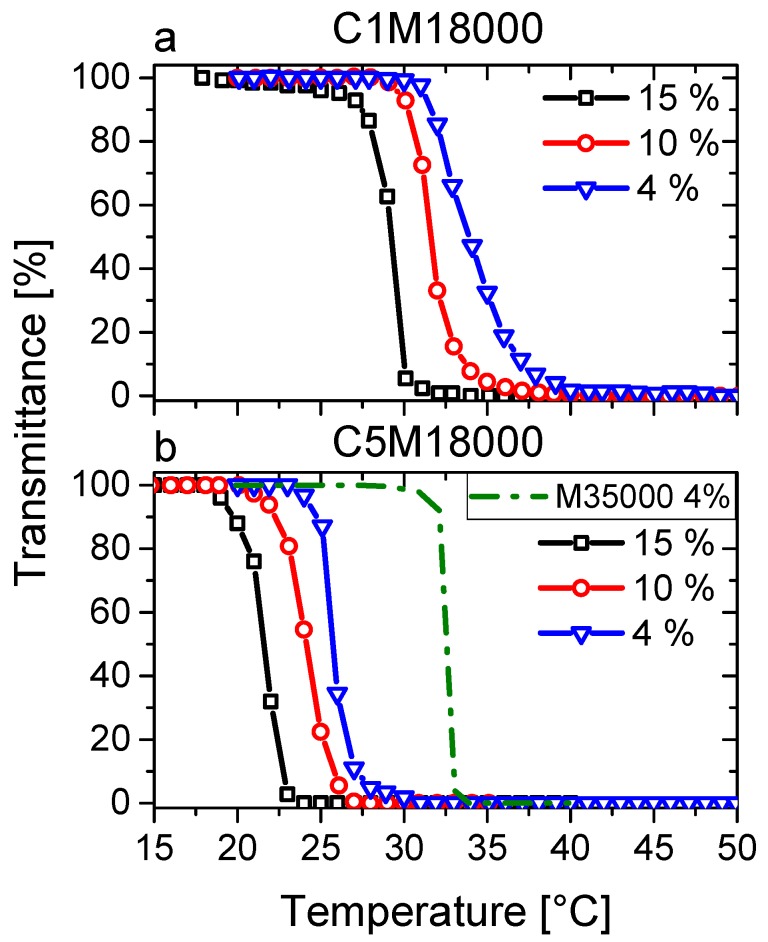
Transmittance as a function of temperature of aqueous solutions (4 wt %, 10 wt %, and 15 wt %) for C1M18000 (**a**) and C5M18000 (**b**) samples.

Figure 5 shows the transmittance curves vs. temperature of the diverse polymer solutions in water (4, 10, and 15 wt %) of C1M18000 (Figure 5a) and C5M18000 (Figure 5b) samples. LCST transitions, studied by transmittance (50%), are in good agreement with the endotherm peaks (Figure 3).

However, here, LCST values describe a decrease with increasing polymer concentration in water, which is observed to a much lower degree in DSC data. The reason for the stronger temperature dependence of the UV-visible spectroscopic data was concluded to be a lower percentage of the dissolved polymer will lead to a 50% transmission loss at higher polymer concentration, which means that the UV-vis data have to be regarded to be less reliable in giving the true LCST temperatures than DSC, due to their different response characteristics.

**Figure 6:**
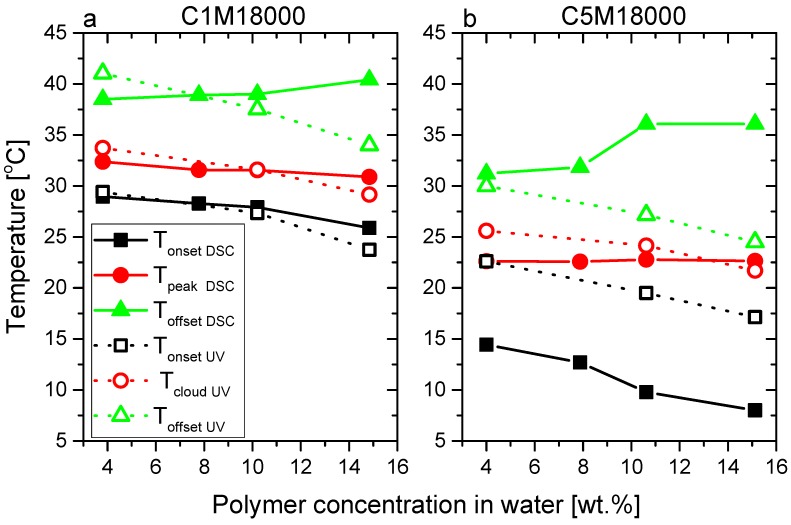
Peak temperatures (T_peakDSC_), cloud points (T_cloudUV_), onset temperatures (T_onsetDSC_ and T_onsetUV_), and offset temperatures (T_offsetDSC_ and T_offsetUV_) derived from calorimetric analysis and transmittance curves, respectively.

The LCST transitions, analyzed from calorimetric curves (Figure 3) and transmittance data (Figure 5), were evaluated not only with respect to the peak point (T_peakDSC_) and the cloud point (T_cloudUV_), defined as the half-height of the transmittance transition, as classically done, but also from the onset and offset temperatures (T_onsetDSC_ and T_onsetUV_/T_offsetDSC_ and T_offsetUV_). The results were collected and displayed in Figure 6, where an interesting equivalence can be deduced from peak temperatures (T_peakDSC_) and cloud points (T_cloudUV_).

Both peak temperatures (T_peakDSC_) and cloud points (T_cloudUV_) exhibit similar values for C1M18000 (Figure 6a) and C5M18000 (Figure 6b), indicating that phase transitions happen in analogous ranges as the endotherms and transmittance data show. Hence, the different heating rates used (5 vs. 1 °C/min) do not play a significant role for the polymeric aqueous solutions from a physicochemical standpoint. Therefore, results can be discussed according to the molecular features and the sample conditions.

The cloud points of C1M18000 ranged between 29 and 33 °C and seem to describe a trend in relation to polymer concentration in water, closely resembling the DSC data (T_peakDSC_). In contrast to this, while the cloud points of 5M18000 are located between 21.5 and 26 °C, again following a concentration-dependent trend, the peak temperatures (T_peakDSC_) display constant values for all concentrations in water. The DSC sensitivity to polymer concentrations in water could depend on the hydrophobic interactions. Nevertheless, calorimetry provides important information of these interactions such as endotherms exhibited for C5M18000.

It is obvious that a difference between T_onsetDSC_ and T_offsetDSC_ exists, which is higher for C5M18000 vs. C1M18000. This effect could be explained according to hydrophobic interactions [32,33,34,35], produced by the presence of DMA in relation to its higher hydrophobicity in comparison with NIPAM [11] promoting long LCST transitions because these interfere along LCST transition, i.e., the aforementioned hydrophobicity fluctuations induced by the statistical distribution of DMA induce a distribution of local hydrophobicity, which will lead to local LCST fluctuations, leading to a broadening of the global LCST process (Figure 4). This is similar to the work on ethylene–hexene copolymers, where a precise placement of the comonomer, e.g., every 15^th^ main chain carbon leads to a significantly more narrow and higher melting point than found for the standard random copolymers [38,39]. Secondly, the T_onsetDSC_ decreases, while T_offsetDSC_ increases when the polymer concentration rises for both samples. The difference between T_onsetDSC_ and T_offsetDSC_ achieves a maximum for the highest concentration of 15 wt %, especially for C5M18000 where a great number of hydrophobic interactions occur.

**Figure 7:**
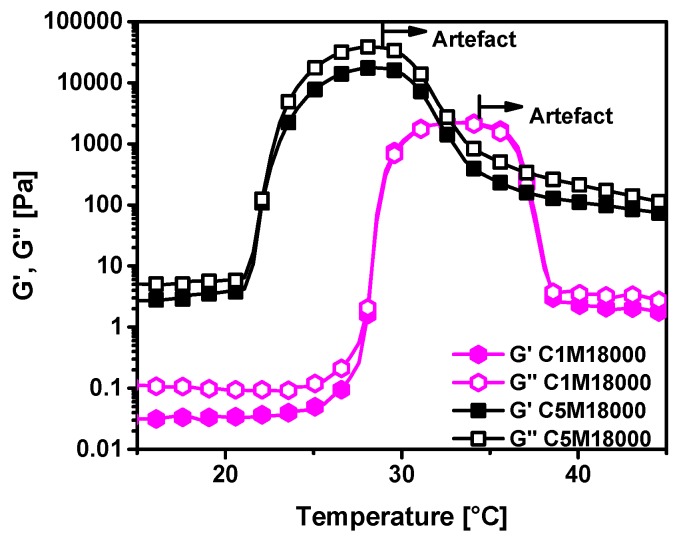
Temperature ramps of homopolymers solutions in water at 15 wt.% for C1M18000 and C5M18000.

Temperature ramps (Figure 7) were tested by rheology for both samples at the same polymer concentration in water of 15 wt %, with both solutions showing a clear increase in storage and loss moduli around the LCST. The LCST transitions are located at almost identical temperatures as those from conventional calorimetry and UV-measurements. Thus, consistent data is achieved from the diverse detection methods used along this research work. The LCST transitions are wider than transitions related to UV-measurements and calorimetric analysis. This fact could be related to rheology, which can detect the primary traces to the coil-to-globule transition earlier than the other techniques and, furthermore, rheology can also detect the offsets while UV-vis cannot find any more increment due to complex opacity of the sample. The reasons for the higher sensitivity of rheology are multifarious. They mainly come from the fact that in general rheology reacts highly sensitively to the slightest changes in sample structure, such as the first traces of globule formation. Those globule onsets are too small to be detected by UV-vis due to their size being below the wavelength of visible light. Furthermore, supposedly the number of H-bonds being formed is too low to produce a clear calorimetric signal.

It should be mentioned that ca. 8 K above the LCST, the data go through a maximum, which is a well-known artefact related to the sample loosing contact with both plates due to its LCST induced contraction [11,40,41]. Consequently, all data at temperatures above this peak should be ignored.

The moduli for C5M18000 are significantly higher than for C1M18000 below the LCST, which can be understood as the consequence of the sample C5M18000 being able to complex with each other through radical crosslinking of the dopamine groups as well as through capturing of ions, which would eventually lead to crosslinking. These ions diffuse away from the surface of the stainless steel plates and are, thus, supposedly mostly iron and nickel ions in nature, which are known to have strong interactions with dopamine groups [42]. As Fe^3+^ ions are known to form di-dopamine complexes with a dirty greenish color, it is not surprising that the sample C5M18000 had a slightly greenish hue at the end of the experiment, which of course does not mean that the sample’s dopamine groups were saturated with ions, but only that a rather small amount of ions diffused in. This can also be seen from the fact that the samples are not gels.

### 3.4. LCST for Copolymers with Different Molecular Weight

**Figure 8:**
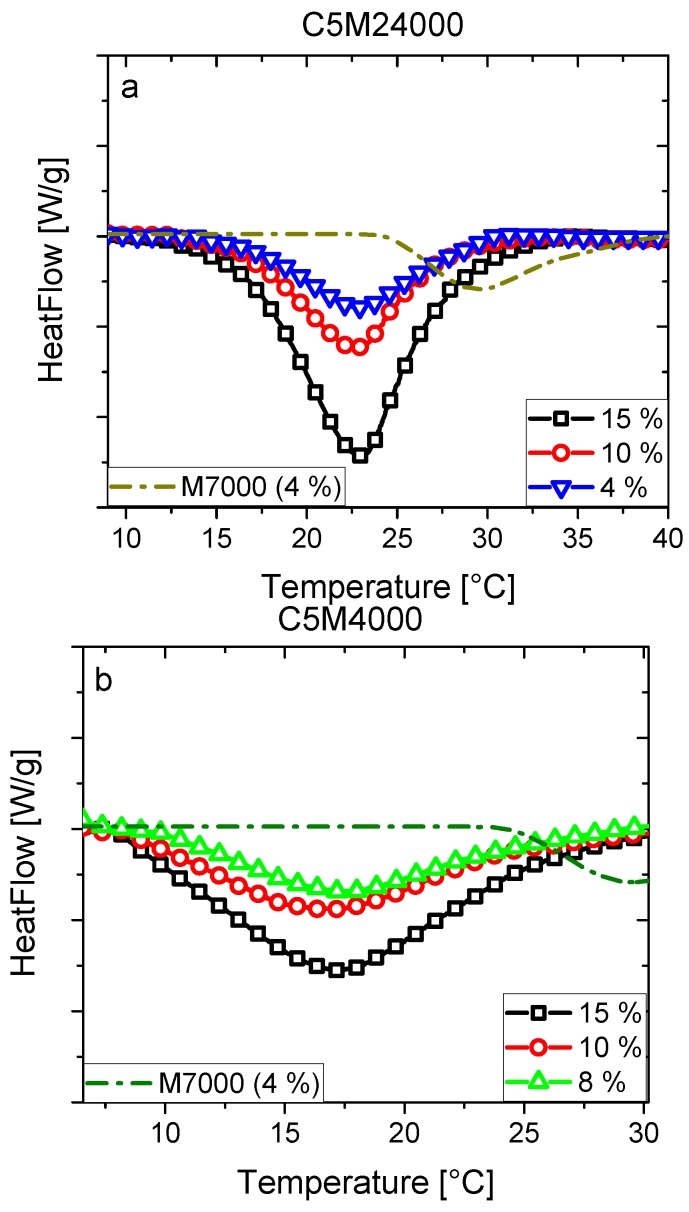
DSC endotherms of aqueous solutions for C5M24000 (**a**) and C5M4000 (**b**) samples. The results were compared with M7000 homopolymer (c = 4 wt %).

C5M24000 and C5M4000 aqueous solutions were studied by conventional calorimetric analysis (Figure 8), and results were also compared with C5M18000. Differences between endotherms were evident due to LCST transitions of C5M4000 were located around to 5 °C below to C5M18000 and C5M24000. Nevertheless, LCSTs were somewhat lower than it could be expected by the results previously reported [11]. In that case, the molecular weight was lower (2800 g/mol), as well as the comonomer content was also somewhat lower (≈4.9 mol %). Nevertheless, some considerations should take account in order to understand the different results.

On the one hand, the previous work reported a copolymer prepared by free radical polymerization, whose polydispersity was 2.1 and whose chain ends were far less hydrophobic. Thus, the presence of the RAFT agent leads to an additional influence on the LCST behavior for C5M24000, C5M18000, and C5M4000. Furthermore, it needs to be taken into consideration that the data were measured by a rheometer, whose temperature calibration might be somewhat different from the one used here. In addition, the polymer concentration was 20 wt % [11] and not max. 15 wt % as studied in this paper.

C5M24000 and C5M18000 endotherms (Figure 3b and Figure 8a) showed higher LCSTs than C5M4000 (Figure 8b) due to a molecular weight increase, leading to a lower hydrophobicity difference as previously discussed by us for PNIPAM [11], because end-groups are reduced and consequently hydrophobic interactions. The M7000 homopolymer exhibited the highest LCST showing the strong effect of the DMA content in C5M24000, C5M18000, and C5M4000. This effect was slightly perceived for the differences between LCSTs of C5M24000 and C5M18000, but the changes between DMA content made difficult extracting conclusions. However, it should also be mentioned that, while the comonomer contents were similar, they were slightly different. In the next section, we will discuss these effects in detail.

Nevertheless, the influence of molecular weight on LCST was discussed along the literature where some inconsistences and contradictions can be observed between trends and behaviors [32]. Some parameters as polymerization degree or end-group effect could significantly contribute on LCST over the molecular weight, and consequently, could explain the different results reported. Thus, it is necessary to carry out a deep study, where the influence of each parameter can be explained separately before reaching conclusions focused only around to molecular weight.

The endotherms of C5M4000 appear more smeared out and wider than transitions from C5M24000 or C5M18000. As said above (Figure 4), the hydrophobic interactions can lead to this kind of LCST transitions, because they are caused by hydrogen bonds cleavage. Nevertheless, very similar endotherms should be expected due to very similar DMA contents. Thus, the presence of the RAFT-agent at the end of the polymer chain must be the responsible of this effect, as the RAFT-agent content increases as polymerization degree decreases promoting the end-group effect.

**Figure 9:**
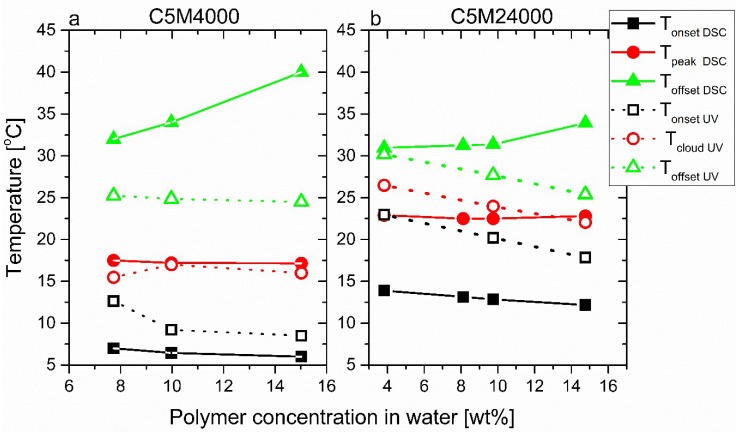
Peak temperatures (T_peakDSC_), cloud points (T_cloudUV_), onset temperatures (T_onsetDSC_ and T_onsetUV_), and offset temperatures (T_offsetDSC_ and T_offsetUV_) derived from calorimetric analysis and transmittance curves for C5M4000 and C5M24000.

The LCST transitions were studied in detail through the estimation of onset, peak, and offset temperatures (T_peakDSC_, T_onsetDSC_, and T_offsetDSC_, analogously to Figure 5) of calorimetric curves for C5M4000 and C5M24000 solutions with different concentrations. Furthermore, some onset, cloud, and offset points (T_cloudUV_, T_onsetUV_, and T_offsetUV_) obtained from transmittance data were included and an excellent correspondence between LCSTs can be observed. In this way, both peak temperatures (T_peakDSC_) and cloud points (T_cloudUV_) exhibit similar values for C5M4000 (Figure 9a) and C5M24000 (Figure 9b).

Again, the C5M24000 aqueous solutions (Figure 9b) show different cloud points between 22.5 °C and 27 °C, whose trend corresponds to the polymer concentration in water. Nevertheless, this tendency is not observed from peak temperatures of calorimetric analysis. Likewise, this trend is not shown for C5M4000, whose T_cloudUV_ and T_peakDSC_ values are around 17 °C, probably due to strong hydrophobic interactions explained before for the copolymers with different DMA amount.

**Figure 10:**
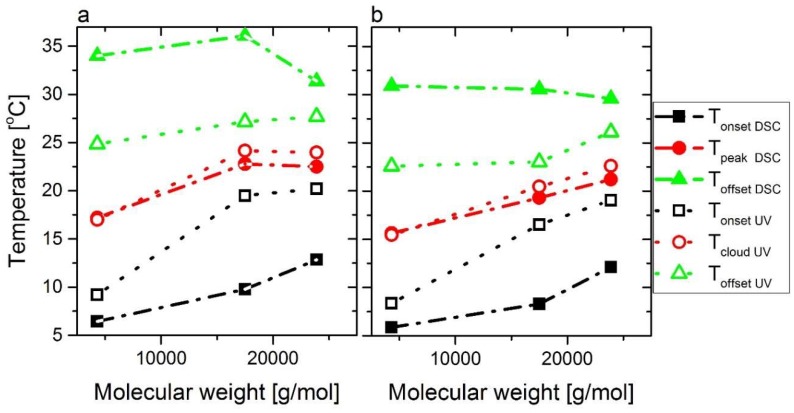
(**a**) Peak temperatures (T_peakDSC_), cloud points (T_cloudUV_), onset temperatures (T_onsetDSC_ and T_onsetUV_), and offset temperatures (T_offsetDSC_ and T_offsetUV_) derived from calorimetric analysis and transmittance curves vs. molecular weights for C5M4000, C5M18000, and C5M24000 at the same polymer concentration in water (10 wt %). (**b**) Same data but normalized to 5 mol % DMA content using a statistical model.

It is obvious from Figure 10a that the differences between onset and offset temperatures increased significantly with decreasing molar mass, which needs to be explained. For this purpose it was determined how many DMA groups are on the different chains, assuming a binomial distribution probability, i.e., that the monomers are incorporated 100% randomly. The result was that for C5M4000 (DP_n_ = 31, end groups’ molar mass 272.42 g/mol) 20.3% of the chains did not have any DMA in them, 33.3% had 1, 26.2% had 2, 13.3% had 3, and 6.7% had more than 3 (the numbers are not entirely correct, as it is assumed that the average molar mass per monomer unit was constant, but that was clearly not the case, as DMA was about twice as heavy as NIPAM and, therefore, chains with fewer DMA groups but the same DP_n_ as those with more DMA groups had a lower molar mass. However, this influence was considered to be minor (estimated to be <±2%) and, therefore, ignored for further discussions). Consequently, there are several species of chains in C5M4000, which differed in their hydrophilicity—most prominently, the ca. 20% of pure PNIPAM in C5M4000 should have a slightly lower LCST than M7000 (ca. 25 °C), while the observed LCST is at 17 °C, ca. 8 K lower. Considering this difference alone, it is logical that such a material has a much broader LCST than a homopolymer material or a copolymer with significantly higher comonomer content. For C5M24000, the six times higher molar mass leads to a negligible homopolymer fraction and 80% of the chains contain 6–13 DMA functionalities, suggesting the inhomogeneity in comonomer distribution is much lower. Furthermore, as pointed out previously, the rather hydrophobic RAFT-agent residues at the chain ends lead to a further gradient in hydrophobicity, which also explains the same kind of effect for the PNIPAM-homopolymers M7000 and M35000. For low molar masses (e.g., C5M4000 has a DP_n_ of only ca. 31), this end group effect plays a significant role, while for C5M24000 (DP_n_ ca. 200) this effect is much less pronounced. These two effects lead to a higher LCST of C5M24000 taking place in a narrower temperature range than C5M4000. Hence, the LCST of C5M24000 has to be considered to be the normal LCST value of copolymer with ca. 5 mol% DMA and the RAFT end groups of C5M4000 lower the LCST due to the end group effect.

This trend seems slightly disturbed by C5M18000 but this fact can be perfectly understood from its higher amount of DMA. Nevertheless, if the data is normalized according to comonomer amount, clear trends in relation to molecular weights will be observed for the LCST derived from calorimetry (T_peakDSC_) and UV-vis spectroscopy (T_cloudUV_) as Figure 10b shows, where the data is normalized for a DMA content of 5 mol %. Moreover, tendencies are defined according to hydrophobic interactions for T_onsetDSC_, and T_offsetDSC_ regarding to T_onsetUV_ and T_offsetUV_. Again, calorimetry seems more sensitive than UV-vis spectroscopy to these hydrophobic transitions, which could partially explain the contributions of the end-group effects on LCST.

**Figure 11:**
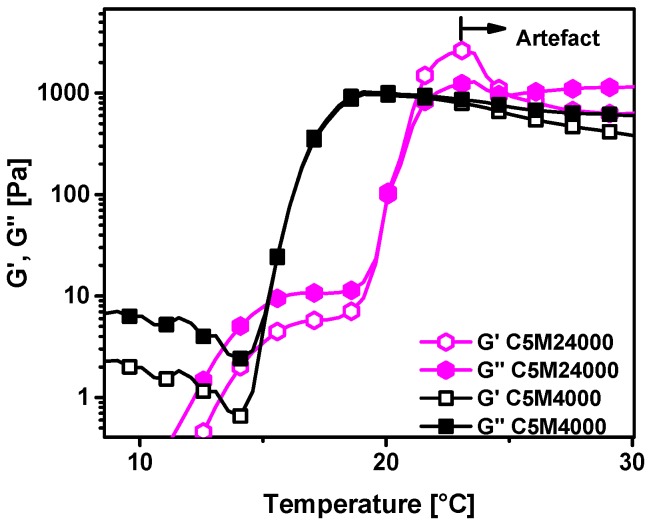
Temperature ramps of homopolymers solutions in water at 10 wt % for C5M4000 and C5M24000.

The rheological properties were tested for both C5M4000 and C5M24000 at the polymer concentration in water of 10 wt % (Figure 11). Again, the LCST transitions show an excellent correspondence with the data collected from conventional calorimetric analysis and turbidimetry.

It should be mentioned that just like in our previous paper, a small shoulder is obvious between 15 and 20 °C for C5M24000, which might be the consequence of high local DMA concentrations along the chain [11]. Otherwise, the curves follow the same pattern and exhibit very similar artefacts as previously discussed.

### 3.5. LCST Ranges

Commonly, LCST is defined as an exact temperature where the transition to coil to globule takes place. Nevertheless, LCST is a process where the hydrogen bonds are breaking according to the influence of various factors discussed before [1,37]. Consequently, the LCST is not occurring at a single temperature, but in a certain interval. Thus, important information can be lost and, consequently, the study of the transition as a whole is for understanding the real influence of some parameters on the LCST.

This work defined the LCST ranges as the difference between offset and onset temperatures (T_onsetDSC_ and T_offsetDSC_) derived from DSC endotherms. While other LCST ranges could be estimated from turbidimetry or rheology, calorimetric analysis could provide an important information because the endotherm is more precise than the other methods and, furthermore, directly related to the hydrogen bonds involved on LCST process [21] and, thus, the data are more related to the molecular processes. As mentioned above, the endotherms could reflect other mechanisms as hydrophobic interactions shown from C5M4000 and C5M24000.

**Figure 12:**
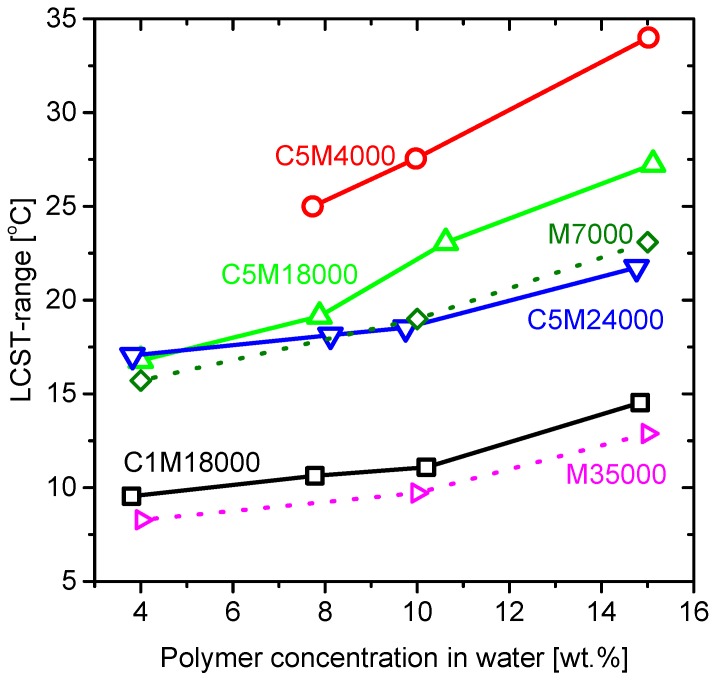
LCST transition ranges versus polymer concentration in water for all samples.

Figure 12 shows the diverse LCST ranges estimated from the different endotherms (Figure 3 and Figure 8) for all the samples and were displayed as a function of polymer concentration in water. The LCST ranges changed in respect of the polymer concentration in water. This effect could be ascribed to the coil-to-globule transition, as less concentrated solutions tend to aggregate and settle easier. On the other hand, a lower percentage of the dissolved polymer could lead to increase the transition temperature at higher polymer concentration. Moreover, the LCST range dependence on concentration of the sample seemed to be connected to the LCST range at high concentrations. When extrapolating the data to zero concentration, the LCST range for all samples appeared to be around 10 °C, suggesting that this was the minimum width observable for the LCST. As C5M4000 had the broadest LCST, it also showed the highest slope regarding to other samples in relation to great hydrophobic interactions and the polymer concentration in water.

Hence, these transitions open a new perspective on LCST behavior because the influence of end-group effect, presence of comonomer and/or molecular weight could be explained through the hydrophobic interactions.

## 4. Statistical Modeling

In order to understand these complex correlations better, a series of regression analyses were conducted to study the influence of the comonomer content (DMA), polymerization degree (DPn) and the polymer concentration in water (wt %; independent variables) on the dependent variables. Those were the parameters involved in the LCST, such as T_onsetDSC_, T_peakDSC_, T_offsetDSC_, and LSCT_rangeDSC_, DSC integral derived from calorimetry, and T_onsetUV_, T_cloudUV_, T_offsetUV_, and LSCT_rangeUV_, obtained by turbidimetry.

In general, the regression analysis model can be explained through equation [1], where the comonomer content (DMA), polymerization degree (DPn), and the polymer concentration in water (wt %) were independent variables, and the parameters involved in the LCST, such as T_onsetDSC_, T_peakDSC_, T_offsetDSC_, and LSCT_rangeDSC_, derived from calorimetry, and T_onsetUV_, T_cloudUV_, T_offsetUV_, and LSCT_rangeUV_, were dependent variables.

Dependent variable (one of the LCST characteristics) = B_0_ + B_1_ (DMA) + B_2_ (DPn) + B_3_ (wt %) (1)

All parameters were displayed in Table 2, were supplemented by the standard error (std. error), t (t should be higher than 1.96 when independent variable has a significant effect on dependent variable), the beta distribution (Beta), and Sig (refers to significance level, which indicates whether independent variable has a significant effect on dependent variable). The regression analysis routine deletes independent variables that are deemed not relevant, which is denoted in Table 2.

The LCST parameters showed interesting results where the influence of comonomer content, polymerization degree and polymeric solution in water was clarified. Firstly, LCST value is general studied by the T_peak_ obtained from the endotherms of calorimetry, and/or the T_cloud_ estimated from turbidimetry, as the next equations show:

T_peakDSC_ = 27.586 °C − 1.617 °C/mol % (DMA) + 0.020 °C/monomer (DPn) (2)

T_cloudUV_ = 20.776 °C − 0.806 °C/mol % (DMA) + 0.042 °C/monomer (DPn) (3)

The results of regression analysis of calorimetry showed that DMA had a negative correlation on T_peak_ (b = −1.617, *p* = 0.000), while DPn had a positive correlation on T_peak_ (b = 0.02, *p* = 0.000), and polymer concentration in water (wt %) had no significant effect on T_peak_. Similarly, the results of regression analysis of turbidimetry showed that DMA was negatively correlated to T_cloud_ (b = −0.806, *p* = 0.000), while DPn had a positive effect on T_cloud_ (b = 0.042, *p* = 0.000), and polymer concentration in water (wt %) had no significant effect on T_peak_. Specifically, the regression analysis of these T_peak_ and T_cloud_ values, as dependent variables, showed that the comonomer content plays an important role in the LCST behavior due to its negative correlation with the LCST value. Consequently, while Figure 6 and Figure 10 seem to suggest a correlation between T_peakDSC_ and T_cloudUV_, the regression analysis clearly shows that DPn and DMA content were significantly more important than the concentration. The reason can be easily understood from the fact that DPn and DMA content change the molecules’ hydrophobicity, while the concentration only brings them somewhat closer or farther apart. The effect of the former was logically much stronger than that of the latter, which does not mean that the concentration does not have an effect—it was just so small that its influence was within the experimental error.

Obviously, the presence of the comonomer decreases the LCST as was reported before and we described it along this work (Figure 6). Nevertheless, the content of DMA seems to influence the LCST detected calorimetry more than turbidimetry, such as could be observed from a higher b_1_ value associated with the calorimetry whose value (−1.617) was double the value related to turbidimetry (−0.806). This fact can be easily understood because turbidimetry measures optical signals and some changes can be produced below the detection range, whilst calorimetry can still detect them. On the other hand, the polymerization degree showed a minor positive correlation with LCST, i.e., the LCST increased with rising polymerization degree. The data shows that the polymerization degree influence on LCST was lower than other parameters, which were responsible of the diverse trends reported and the discussion in the literature.

**Figure 13:**
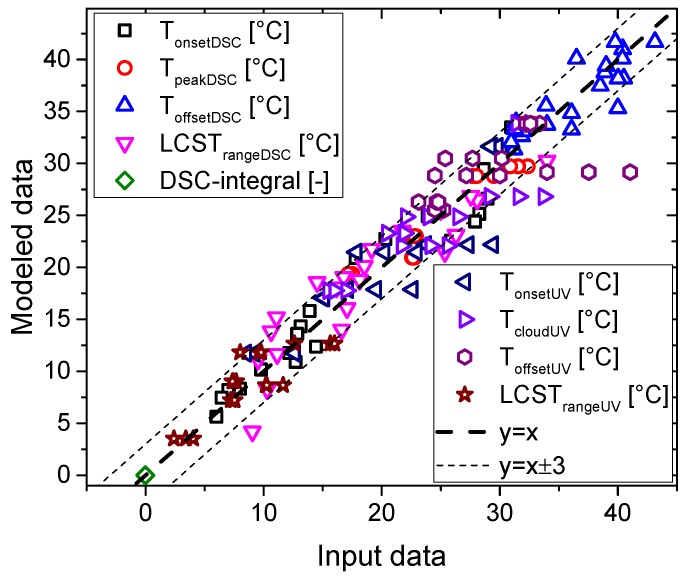
Comparison of input data and modeled data.

In order to visualize the quality of the fits, Figure 13 shows a comparison between input data and the modeled prediction. The thick dashed line represents an ideal correlation, while the thin dashed lines indicate a deviation by ±3, which encompasses almost all results. The r² value of all data in Figure 13 was found to be 0.95598, suggesting all data can be modeled well. Furthermore, it becomes obvious that the correlations for the DSC derived quantities were more precise, especially for the T_peak._ This can be explained from firstly, the cleaner calorimetric signal, especially for the peak, and secondly from the much higher data density of calorimetry with ca. 100 data per K, while only ca. 1 point could be measured per K for UV-vis spectroscopy.

The LCST range was also analyzed using the statistical method for calorimetry and turbidimetry data. The results associated with calorimetry displayed a positive influence of the comonomer content on LCST range, a lower effect of the polymer solution in water, and finally, a small negative effect of polymerization degree was observed from the data obtained by calorimetry. Nevertheless, the data acquired by turbidimetry exclusively showed the negative influence of the polymerization degree on the range LCST range as shown in Table 2. Thus, calorimetry seems more sensitive to the LCST transition than turbidimetry for these materials, i.e., endotherms contained a lot of information of all transition due to the main parameters associated with the molecular features of the samples and the conditions of the samples involved. This fact is really interesting, because the LCST ranges were not deeply studied previously, which we concluded to be the consequence of the majority of the LCST studies being carried out by turbidimetry, where LCST ranges did not seem to be affected by the diverse parameters involved.

Onset and offset temperatures, derived from calorimetry and turbidimetry, were also examined by this statistical method. The calorimetry data showed an interesting relation with onset and offset temperatures through the comonomer content, the polymerization degree and the polymer solution in water. Both temperatures were negatively correlated with DMA content and polymer content in water, but positively influenced by polymerization degree. Nevertheless, less information was obtained by turbidimetry, especially for the offset temperature where polymerization degree effect was exclusively significant.

**Figure 14:**
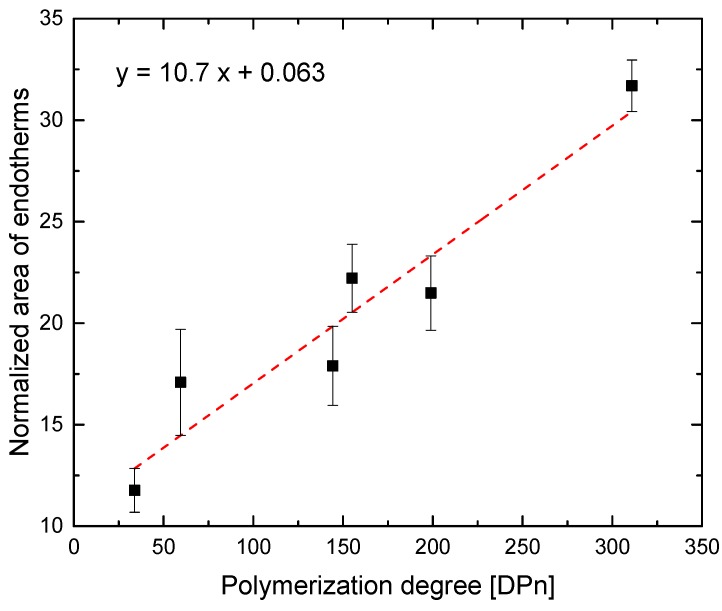
Comparison between polymerization degree vs. normalized are of the endotherms obtained from calorimetry data (dots) and from the simulation (line).

Another statistical study was also performed using the areas obtained from the endotherms of DSC studies (Figure 14), which were previously normalized to the polymer concentration in water and averaged by concentration. A clear linear regression can be observed from these results, where the error bars were estimated through the comparison between experimental data and regression model. The results clearly exhibited a dependence between polymerization degree and the area of the endotherms, as those could be associated with the number of hydrogens involved in the LCST [21]. Obviously, the comonomer content will not affect the final trend so much, because the number of comonomer units along the polymeric branches was rather low, as all the samples present DMA contents below 6%.

In general, statistical modeling can predict the LCST behavior of DSC results according to the molecular features (DMA content and DPn) and the polymer concentration in water. Onset temperatures could exhibit the strongest dependence on those, as these parameters play an important role during the first stages of the LCST transition, especially the comonomer content, which could disrupt the starting point of LCST transition. Further, peak temperature has a similar influence, but the offset temperatures do not seem intensively affected by these factors. Furthermore, the LCST range can exhibit a good resolution, as the onset temperature plays an important role in that value.

## 5. Conclusions

RAFT polymerization allowed for obtaining interesting copolymers to study the effect of the comonomer content and the molecular weight on LCST. A good agreement was observed for the results obtained from calorimetry, turbidimetry, and rheology.

The content of more hydrophobic DMA decreased the LCST for the copolymers, where the comonomer amount was varied. Influence of other parameters, such as polydispersity and the end-group effect were found to be negligible in line with molecular features owing to the high molar mass of those samples.

LCST transitions derived from calorimetric analysis showed a great sensitivity to the change in hydrophobic interactions promoting to wider and more smeared out LCST endotherms, which was quantified in terms of LCST range. Hence, LCST transitions can clearly show the contributions from various molecular parameters in relation to hydrophobic interactions improving the reported analysis just focused on a simple value.

The LCSTs clearly showed the end-group effect for low molar masses, related to the presence of the hydrophobic RAFT-agent, which can be understood as the weight fraction of the RAFT-agent fragments residing at the chain ends decreases with increasing polymerization degree. The LCST ranges increase for low polymerization degrees and increasing concentration. While the influence of polymerization degree and comonomer content are clearly related to the inhomogeneity of the polymer chain (RAFT-residues and DMA leads to locally increased hydrophobicity), the influence of the concentration is probably due to an increasing tendency to phase separate into more or less hydrophobic chain segments with increasing concentration. The driving force behind this kind of process is that at higher concentrations, the statistical probability of chain segments of like hydrophobicity meets each other with a higher probability. That allows for local LCSTs in a wider temperature range.

The statistical model showed that the LCST behavior could be directly related to the comonomer content, polymerization degree and polymer solution in water with an accuracy of less than ±3 K. The statistical modeling allowed for discerning the different contributions of these factors on LCST.

The study of the complete transition associated with LCST could elucidate the diverse and partially contradictory results reported in literature in respect to the influence of parameters involved on LCST. Furthermore, LCST ranges could be very useful for new applications as biosensors or drug carriers where the control of the response will be essential.

Lastly, the application of a statistical model, frequently used in humanities and economics, was able to discern the influences on the different LCST parameters, which could prove a very useful strategy for many cases, where mixed influences prevail or where the trends are not as clear and particularly, where the synthesis or production of exactly defined model systems is not feasible.

## Figures and Tables

**Table 1:** Data of reversible addition-fragmentation chain transfer (RAFT)-polymerization runs and molecular features.

Samples	Monomer Feed Ratio	DMA	M_n_ *	PDI (M_w_/M_n)_ *	T_g_
		mol %	g/mol	-	°C
**M7000**	-	-	7000	1.12	131.4
**C5M4000**	0.06	5.5	4300	1.16	129.0
**C5M24000**	0.06	5.3	23,900	1.45	137.6
**C1M18000**	0.0075	0.6	17,900	1.49	134.8
**C5M18000**	0.06	5.9	17,500	1.44	132.1
**M35000**	-	-	35,400	1.25	137.7

* The molecular weight (Mn) and polydispersity (PDI) were determined from gel permeation chromatography calibrated with narrow molar mass distribution polystyrenes.

**Table 2:** Statistic data obtained for the analysis of the influence of comonomer content (DMA), polymerization degree (DPn), and the polymer concentration in water (wt %).

	Output	Calorimetry				UV-Spectroscopy			
Input		T_onsetDSC_	T_peakDSC_	T_offsetDSC_	LSCT_rangeDSC_	T_onsetUV_	T_cloudUV_	T_offsetUV_	LSCT_rangeUV_
**Constant**	**B_0_**	24.374	27.586	36.907	13.608	13.607	20.776	24.508	13.783
	**Std. Error**	1.712	0.943	1.263	2.318	1.975	1.791	2.008	0.924
	**Beta**	-	-	-	-	-	-	-	-
	**t**	14.241	29.245	29.226	5.871	6.89	11.601	12.208	14.916
	**Sig**	0	0	0	0	0	0	0	0
**DMA**	**B_1_**	−2.618	−1.617	−1.157	1.417	−0.696	−0.806	n.s.	n.s.
	**Std. Error**	0.182	0.139	0.169	0.246	0.305	0.276	n.s.	n.s.
	**Beta**	−0.813	−0.821	−0.807	0.547	−0.277	−0.384	n.s.	n.s.
	**t**	−14.401	−11.64	−6.86	5.756	−2.285	−2.917	n.s.	n.s.
	**Sig**	0	0	0	0	0.037	0.011	n.s.	n.s.
**DPn**	**B_2_**	0.034	0.02	n.s.	−0.039	0.058	0.042	0.03	−0.033
	**Std. Error**	0.006	0.004	n.s.	0.008	0.009	0.008	0.011	0.005
	**Beta**	0.333	0.33	n.s.	−0.483	0.774	0.682	0.551	−0.845
	**t**	5.889	4.685	n.s.	−5.077	6.391	5.176	2.643	−6.321
	**Sig**	0	0	n.s.	0	0	0	0.018	0
**Polymer concen-tration in water**	**B_3_**	−0.367	-	0.319	0.678	n.s.	n.s.	n.s.	n.s.
	**Std. Error**	0.117	-	0.112	0.159	n.s.	n.s.	n.s.	n.s.
	**Beta**	−0.172	-	0.336	0.395	n.s.	n.s.	n.s.	n.s.
	**t**	−3.135	-	2.856	4.275	n.s.	n.s.	n.s.	n.s.
	**Sig**	0.006	-	0.01	0.001	n.s.	n.s.	n.s.	n.s.

n.s. = not significant.

## Supplementary Materials

The supplementary materials are available online at https://www.mdpi.com/2073-4360/11/6/991/s1.
