# Supplementary material for: Effect of Hydrophobic Interactions on Lower Critical Solution Temperature for Poly(N-isopropylacrylamide-co-dopamine Methacrylamide) Copolymers
Source: Polymers (Basel). 2019 Jun 4;11(6):991. doi: 10.3390/polym11060991 (PMC6630648; doi:10.3390/polym11060991)
Supplement: Supplementary file 1 [file polymers-11-00991-s001.pdf]

SUPPORTING INFORMATION

Effect of hydrophobic interactions on lower critical solution temperature for poly(N-isopropylacrylamide-co-dopamine methacrylamide) copolymers

Alberto García-Peñas,[1,2] Chandra Sekhar Biswas,[1] Weijun Liang[1], Yu Wang,[1] Pianpian Yang,[3,] Florian J. Stadler[1,*]*

[1] College of Materials Science and Engineering, Shenzhen Key Laboratory of Polymer Science and Technology, Guangdong Research Center for Interfacial Engineering of Functional Materials, Nanshan District Key Laboratory for Biopolymers and Safety Evaluation, Shenzhen University, Shenzhen, 518055, PR China

[2] Key Laboratory of Optoelectronic Devices and Systems of Ministry of Education and Guangdong Province, College of Optoelectronic Engineering, Shenzhen University, Shenzhen 518060, P. R. China

[3] Department of Management , Shenzhen University, Shenzhen 518060, Guangdong, P.R. China.

ORCID: AGP 0000-0001-5707-0198, CSB 0000-0001-6060-890, WJL 0000-0002-8785-3510, YW 0000-0002-2830-5442, PY 0000-0001-6500-5791, FJS 0000-0002-5849-1485

Correspondence to:

Prof. Florian Stadler
College of Materials Science and Engineering, Shenzhen University, Shenzhen 518055, P. R. China
Email: fjstadler@szu.edu.cn
Phone: +86-0755-8671-3986

Prof. Pianpian Yang, Department of Management, Shenzhen University, Shenzhen 518060, P. R. China; Email: yangpianpian2008@hotmail.com; Phone: +86-0755-8671-3986

Structure of copolymers

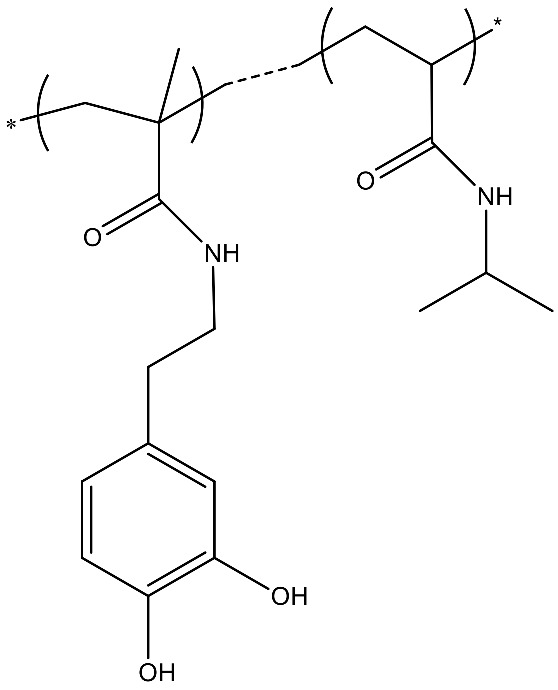

SIScheme 1. Structure of random copolymers.

^{1}H-NMR spectra

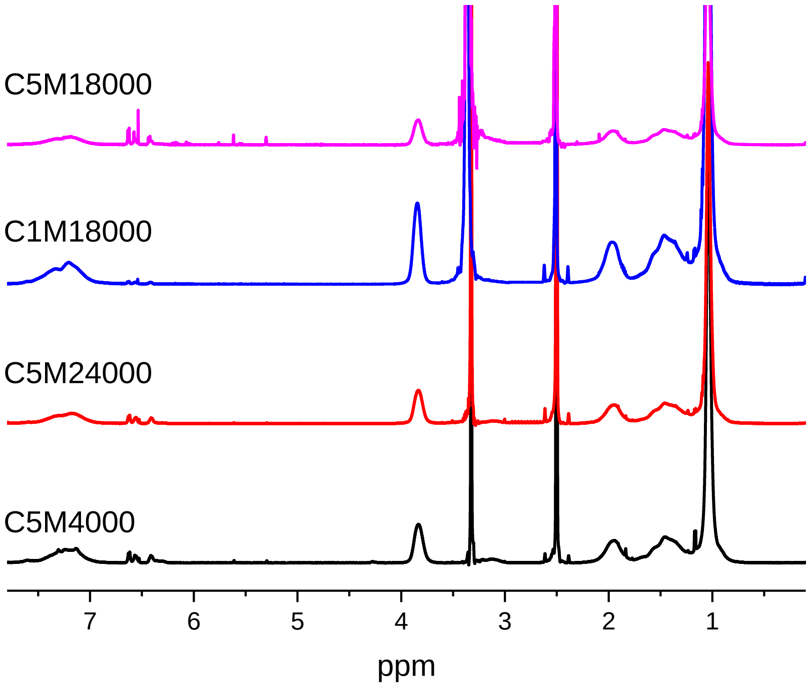

SIFigure 1. ^{1}H-NMR spectra of poly(N-isopropylacrylamide-*co*-dopamine methacrylamide) copolymers.

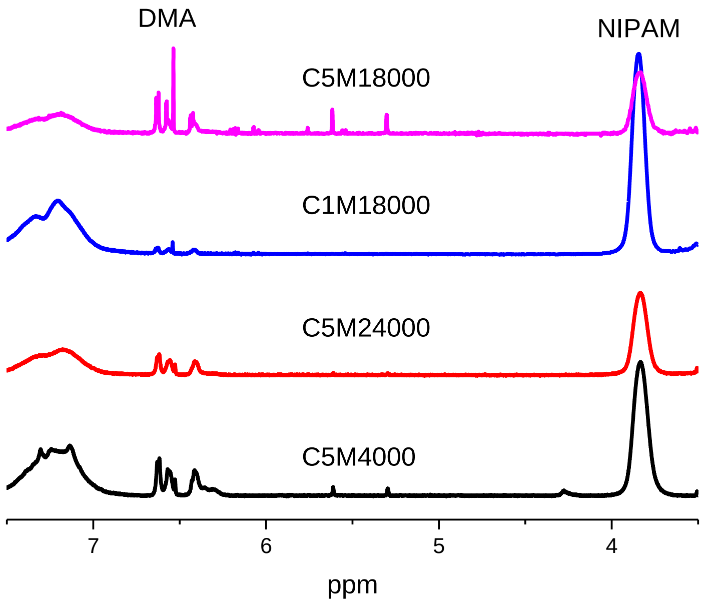

SIFigure 2. ^{1}H-NMR: Identification of the area for quantification.

Analysis of real concentrations (TGA-data)

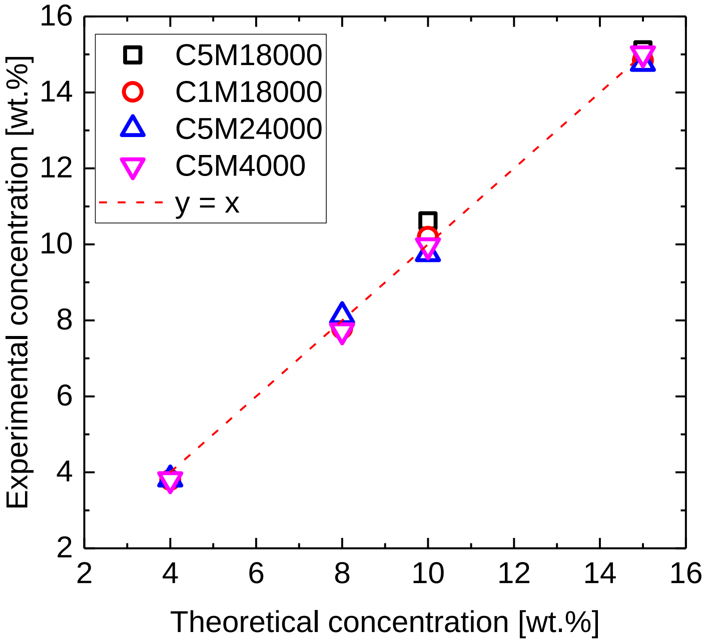

SIFigure 3. Theoretical concentrations of polymeric solutions in water vs. experimental concentrations estimated by TGA-analysis.
